# Editorial: Methods and interventions to predict and tackle suicide risk

**DOI:** 10.3389/fpsyt.2024.1431408

**Published:** 2024-07-01

**Authors:** David Benrimoh, Shannon Lange, Tihare Zamorano, Timothy Friesen, Demián Emanuel Rodante

**Affiliations:** ^1^ Douglas Research Center, Montreal, QC, Canada; ^2^ Department of Psychiatry, McGill University, Montreal, QC, Canada; ^3^ Institute for Mental Health Policy Research, Centre for Addiction and Mental Health, Toronto, ON, Canada; ^4^ Campbell Family Mental Health Research Institute, Centre for Addiction and Mental Health, Toronto, ON, Canada; ^5^ Department of Psychiatry, University of Toronto, Toronto, ON, Canada; ^6^ Institute of Medical Science, University of Toronto, Toronto, ON, Canada; ^7^ University of British Columbia Therapeutics Initiative, Vancouver, BC, Canada; ^8^ Facultad de Medicina, Universidad de Buenos Aires, Instituto de Farmacología, Buenos Aires, Argentina; ^9^ “Dr. Braulio A. Moyano” Neuropsychiatric Hospital, Buenos Aires, Argentina; ^10^ FORO Foundation for Mental Health, Buenos Aires, Argentina

**Keywords:** suicidality, suicide prediction, model bias, suicide, suicide prevention

Suicide is a global public health problem, with a long history of research at both the clinical and population health level. Suicide research is largely driven by the idea that it is preventable (1, 2). A great deal of training of mental health practitioners is spent on techniques aimed at assessing suicide risk, based on risk factors in the hope of predicting and preventing suicide (3, 4). Unfortunately, the reality is that predicting suicide is extremely challenging clinically (5, 6). As such, novel approaches to predicting and tackling suicide risk at both the population and clinical level are needed–a challenge accepted by the authors of the articles in this special edition.

Researchers continue the important work of identifying risk factors for suicide, and demonstrate how they can be incorporated into a variety of risk prediction models. Petho et al. provide evidence that inflammatory markers may be an important biological correlate of suicide risk. This work builds on the literature identifying biological factors associated with suicide, such as genetics and hypothalamic-pituitary axis dysfunction. While not directly actionable at present in most clinical settings, biological insights increase the chance of mechanistic breakthroughs, which may lead to novel treatment or prevention strategies.

In their contribution predicting suicide risk among Colombian students, Narvaez et al. identified some risk factors that may be common across populations (e.g. family history of suicide or exposure to trauma or substances), as well as other factors that may be population-specific, such as being a student who has travelled in order to attend university. Narvaez and colleagues’ findings suggest that further ‘fine tuning’ of these models on population-specific characteristics may be necessary (7). This sheds light on the need to conduct new, higher-quality research in regions that contribute a large percentage to the global suicide rate, such as low- and middle-income countries (8).

Bandara et al. explored using risk factors in intervention by determining the population-attributable fraction of suicide in order to identify which subpopulations, if their risk factors were modified, would experience the greatest overall reductions in suicide. This approach reverses the usual method of deriving risk factors from individual data and then using these to identify potentially at-risk populations and instead looks at determining which subpopulations are at greatest risk of suicide. This method may be helpful in identifying subpopulations at risk who may not otherwise receive the same amount of attention as those traditionally considered at risk of suicide.

The contribution of Tio et al. ties the above papers together, by presenting a systematic review of machine learning studies using a combination of biological (e.g. sleep, HPA axis measures), psychological (e.g. symptom) and social (e.g. marital status) markers to predict suicide-related outcomes. This study reminds us of the potential value of combining markers using a biopsychosocial model to optimize predictions.

Somé et al. present a more general review of machine learning model predictions of different suicide-related outcomes. Encouragingly, they find that, on some metrics, these models perform well, often demonstrating area under the receiver operating curve values of greater than 0.8. However, their review also raises some important concerns for the field. Firstly, they noted that the most important predictors for suicide-related outcomes varied depending on the data source and modelling technique used. This dataset specificity may be caused by unstandardized variable collection. This means that external replication of models between datasets may be challenging, and that an optimal set of predictors may remain elusive with the data currently available. In addition, they found that the mean positive predictive value (PPV), across models, was 0.412. This metric represents the proportion of patients classified as at-risk who will actually experience the negative outcome. This is a crucial metric for suicide prediction models if they are to be applied at the individual level: deciding a patient is at risk for suicide has significant implications, ranging from increased intervention effort to preventative confinement, at the extreme. Implementing these models in practice will require careful ethical consideration (9). Finally, Somé et al. note that the majority of machine learning studies are conducted in Western nations. Variability in optimal predictors occurs within these studies, which further underscores the findings of Narvaez et al., which indicate the importance of localizing models prior to use in order to ensure they are effective within the target population.

This focus on implementation is exemplified in the contribution by Minian et al. Echoing the work of Bandara et al., they select one population known to be at risk of suicide, smokers. They detail their approach to the implementation of a suicide reduction intervention in primary care using a well-known change management technique: plan-do-study-act cycles. They discuss important solutions to common challenges in implementing suicide prevention (such as improving links between primary and mental healthcare). Their work reminds us that even the most accurate predictive model will never help a single patient unless it is linked to effective interventions which can be feasibly implemented into clinical practice.

Overall, the articles in this Research Topic present a series of key messages for the field of suicide prediction and prevention. The first is that, due to the variability in predictors selected by models across datasets, there remains significant value in research focusing on identifying both general and population-specific risk factors. This literature allows us to interrogate models generated using machine learning (10, 11). The second is that machine learning models perform well on some key metrics (12) but will likely require health systems to do the hard work of data harmonization– likely driven by the literature on risk factors– to produce large datasets, which will allow for further model optimization. Further work on models personalized to individual patients could offer insight into managing population heterogeneity (13, 14). Thirdly, even optimized models may benefit from localization, taking advantage of locally-important risk factors to improve performance. Finally, those creating predictive models of suicide-related outcomes must think ahead to how their models will be paired with interventions and implemented in order to generate positive outcomes for patients.

## Author contributions

DB: Conceptualization, Supervision, Writing – original draft, Writing – review & editing. SL: Conceptualization, Writing – original draft, Writing – review & editing. TZ: Writing – review & editing. TF: Writing – review & editing. DER: Conceptualization, Writing – original draft, Writing – review & editing.
